# Ethyl 2-amino-4,6-bis­(4-fluoro­phen­yl)cyclo­hexa-1,3-diene-1-carboxyl­ate

**DOI:** 10.1107/S160053681200373X

**Published:** 2012-02-04

**Authors:** Jerry P. Jasinski, James A. Golen, S. Samshuddin, B. Narayana, H. S. Yathirajan

**Affiliations:** a Department of Chemistry, Keene State College, 229 Main Street, Keene, NH 03435-2001, USA; b Department of Studies in Chemistry, Mangalore University, Mangalagangotri 574 199, India; c Department of Studies in Chemistry, University of Mysore, Manasagangotri, Mysore 570 006, India

## Abstract

In the title compound, C_21_H_19_F_2_NO_2_, the cyclo­hexa-1,3-diene ring is in a distorted envelope conformation. The dihedral angles between the mean planes of the diene moiety and the two fluoro­phenyl rings are 42.8 (2) and 75.0 (5)°. The two fluoro­phenyl rings are inclined to one another by 87.0 (3)°. In the crystal, intra­molecular N—H⋯O hydrogen bonds and weak N—H⋯O and N—H⋯F inter­molecular inter­actions are observed forming an infinite two-dimensional network along [011].

## Related literature

For background to the applications of cyclo­hexenones, see: Padmavathi *et al.* (1999 ▶, 2000 ▶); Padmavathi, Sharmila, Balaiah *et al.* (2001 ▶); Padmavathi, Sharmila, Somashekara Reddy & Bhaskar Reddy (2001 ▶). For the structure of the precursor of the title compound, see: Dutkiewicz *et al.* (2011 ▶). For various derivatives of 4,4-difluoro­chalcone, see: Fun *et al.* (2010*a* ▶,*b* ▶); Jasinski *et al.* (2010*a* ▶,*b* ▶). For puckering parameters, see: Cremer & Pople (1975 ▶).
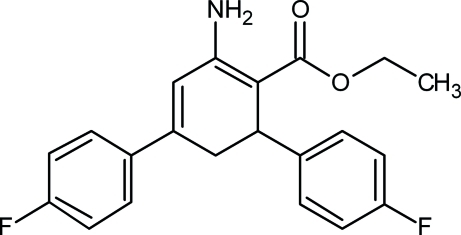


## Experimental

### 

#### Crystal data

C_21_H_19_F_2_NO_2_
*M* *_r_* = 355.37
Orthorhombic, 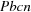

*a* = 18.0199 (5) Å
*b* = 9.6391 (2) Å
*c* = 21.0754 (7) Å
*V* = 3660.70 (18) Å^3^
*Z* = 8
Cu *K*α radiation
μ = 0.80 mm^−1^
*T* = 173 K
0.20 × 0.14 × 0.12 mm

#### Data collection

Oxford Xcalibur Eos Gemini diffractometer
Absorption correction: multi-scan (*CrysAlis RED*; Oxford Diffraction, 2010 ▶) *T*_min_ = 0.856, *T*_max_ = 0.910
10556 measured reflections
3461 independent reflections
2612 reflections with *I* > 2σ(*I*)
*R*_int_ = 0.017

#### Refinement

*R*[*F* ^2^ > 2σ(*F* ^2^)] = 0.055
*wR*(*F* ^2^) = 0.180
*S* = 1.04
3461 reflections
245 parameters
H atoms treated by a mixture of independent and constrained refinement
Δρ_max_ = 0.41 e Å^−3^
Δρ_min_ = −0.21 e Å^−3^

### 

Data collection: *CrysAlis PRO* (Oxford Diffraction, 2010 ▶); cell refinement: *CrysAlis PRO*; data reduction: *CrysAlis RED*; program(s) used to solve structure: *SHELXS97* (Sheldrick, 2008 ▶); program(s) used to refine structure: *SHELXL97* (Sheldrick, 2008 ▶); molecular graphics: *SHELXTL* (Sheldrick, 2008 ▶); software used to prepare material for publication: *SHELXTL*.

## Supplementary Material

Crystal structure: contains datablock(s) global, I. DOI: 10.1107/S160053681200373X/mw2046sup1.cif

Structure factors: contains datablock(s) I. DOI: 10.1107/S160053681200373X/mw2046Isup2.hkl

Supplementary material file. DOI: 10.1107/S160053681200373X/mw2046Isup3.cml

Additional supplementary materials:  crystallographic information; 3D view; checkCIF report

## Figures and Tables

**Table 1:** Hydrogen-bond geometry (Å, °)

*D*—H⋯*A*	*D*—H	H⋯*A*	*D*⋯*A*	*D*—H⋯*A*
N1—H1*B*⋯O2^i^	0.86 (3)	2.23 (3)	3.066 (2)	165 (2)
N1—H1*A*⋯O2	0.88 (2)	2.06 (2)	2.708 (2)	130.3 (19)
N1—H1*A*⋯F1^ii^	0.88 (2)	2.37 (2)	3.104 (2)	141.9 (19)

Symmetry codes: (i) 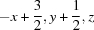; (ii) 
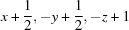
.

**Fig. 1.**
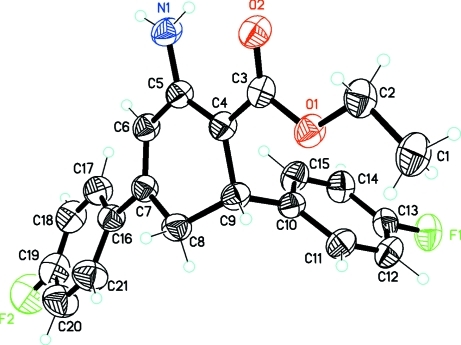
Molecular structure of the title compound showing the atom labeling scheme and 50% probability displacement ellipsoids.

**Fig. 2.**
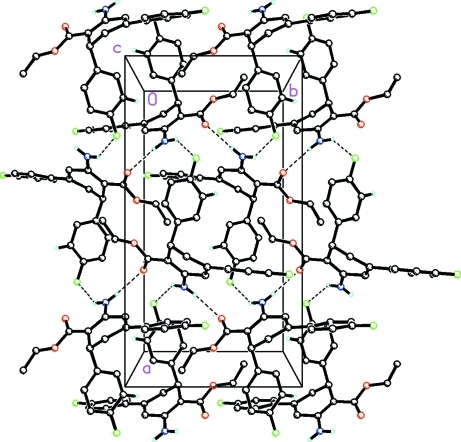
Packing diagram of the title compound viewed along the c axis. Dashed lines indicate N—H···O intramolecular hydrogen bonds and weak N—H···O, N—H···F intermolecular interactions forming an infinite 2-D network along [011]. Remaining H atoms have been removed for clarity.

## supplementary crystallographic information

### Comment

Cyclohexenones are efficient synthons in building spiro compounds (Padmavathi, Sharmila, Somashekara Reddy & Bhaskar Reddy, 2001) or intermediates in the synthesis of benzisoxazoles or carbazole derivatives (Padmavathi *et al.*, 1999, 2000; Padmavathi, Sharmila, Balaiah *et al.*, 2001). The cyclohexenone derivative of 4,4-difluorochalcone reacts with ammonium acetate to yield the title compound (I). The crystal structure of (1RS,6SR)-ethyl 4,6-bis(4-fluorophenyl)-2-oxocyclohex-3-ene-1-carboxylate, which is the precursor of the title compound (I), has been reported (Dutkiewicz *et al.*, 2011). In continuation of our work on the synthesis of various derivatives of 4,4-difluorochalcone (Fun *et al.*, 2010*a*,*b*; Jasinski *et al.*, 2010*a*,*b*), the title compound, (I), was synthesized and its crystal structure is reported here.

In the title compound, C_21_H_19_F_2_NO_2_, the 1,3-cyclohexadiene ring is in a distorted envelope conformation with Cremer & Pople puckering parameters Q, θ and φ of 0.389 (2) Å, 115.8 (3)° and 90.9 (4)° (Cremer & Pople, 1975). For an ideal envelope conformation θ and φ are 54.7° and 120°. The dihedral angles between the mean planes of the diene moiety (C4/C3/O2/O1) and the two fluorophenyl rings are 42.8 (2)° and 75.0 (5)°, respectively (Fig. 1). The two fluorophenyl rings are inclined to one another by 87.0 (3)°. Intramolecular N—H···O hydrogen bonds and weak N—H···O, N—H···F intermolecular interactions (Table 1) are observed forming an infinite 2-D network along [011] (Fig. 2).

### Experimental

A mixture of ethyl 4,6-bis(4-fluorophenyl)-2-oxocyclohex-3-ene-1-carboxylate (3.55 g, 0.01 mol) and ammonim acetate (0.77g, 0.01 mol) in 20 ml of ethanol was refluxed for 10 h. The reaction mixture was cooled and poured into 50 ml of ice-cold water. The precipitate was collected by filtration and purified by recrystallization from ethanol. Single crystals were grown from DMF by the slow evaporation method and the yield of the compound was 70%. (m.p. 428 K).

### Refinement

H1A and H1B were located by a difference map and refined isotropically. All of the remaining H atoms were placed in their calculated positions and then refined using the riding model with atom—H lengths of 0.95 Å (CH), 0.99 Å (CH_2_) or 0.98 Å (CH_3_). Isotropic displacement parameters for these atoms were set to 1.2 (CH, CH_2_) or 1.5 (CH_3_) times *U*_eq_ of the parent atom.

### Crystal data

**Table:**

C_21_H_19_F_2_NO_2_	*F*(000) = 1488
*M**_r_* = 355.37	*D*_x_ = 1.290 Mg m^−^^3^
Orthorhombic, *P**b**c**n*	Cu *K*α radiation, λ = 1.54178 Å
Hall symbol: -P 2n 2ab	Cell parameters from 4418 reflections
*a* = 18.0199 (5) Å	θ = 3.2–71.3°
*b* = 9.6391 (2) Å	µ = 0.80 mm^−^^1^
*c* = 21.0754 (7) Å	*T* = 173 K
*V* = 3660.70 (18) Å^3^	Block, yellow
*Z* = 8	0.20 × 0.14 × 0.12 mm

### Data collection

**Table:**

Oxford Xcalibur Eos Gemini diffractometer	3461 independent reflections
Radiation source: Enhance (Cu) X-ray Source	2612 reflections with *I* > 2σ(*I*)
Graphite monochromator	*R*_int_ = 0.017
Detector resolution: 16.1500 pixels mm^-1^	θ_max_ = 71.4°, θ_min_ = 4.2°
ω scans	*h* = −21→16
Absorption correction: multi-scan (*CrysAlis RED*; Oxford Diffraction, 2010)	*k* = −11→11
*T*_min_ = 0.856, *T*_max_ = 0.910	*l* = −22→25
10556 measured reflections	

### Refinement

**Table:**

Refinement on *F*^2^	Secondary atom site location: difference Fourier map
Least-squares matrix: full	Hydrogen site location: inferred from neighbouring sites
*R*[*F*^2^ > 2σ(*F*^2^)] = 0.055	H atoms treated by a mixture of independent and constrained refinement
*wR*(*F*^2^) = 0.180	*w* = 1/[σ^2^(*F*_o_^2^) + (0.1072*P*)^2^ + 0.4506*P*] where *P* = (*F*_o_^2^ + 2*F*_c_^2^)/3
*S* = 1.04	(Δ/σ)_max_ < 0.001
3461 reflections	Δρ_max_ = 0.41 e Å^−^^3^
245 parameters	Δρ_min_ = −0.21 e Å^−^^3^
0 restraints	Extinction correction: *SHELXL97* (Sheldrick, 2008), Fc^*^=kFc[1+0.001xFc^2^λ^3^/sin(2θ)]^-1/4^
Primary atom site location: structure-invariant direct methods	Extinction coefficient: 0.0010 (2)

### Special details

**Table:**

Geometry. All esds (except the esd in the dihedral angle between two l.s. planes) are estimated using the full covariance matrix. The cell esds are taken into account individually in the estimation of esds in distances, angles and torsion angles; correlations between esds in cell parameters are only used when they are defined by crystal symmetry. An approximate (isotropic) treatment of cell esds is used for estimating esds involving l.s. planes.
Refinement. Refinement of *F*^2^ against ALL reflections. The weighted *R*-factor *wR* and goodness of fit *S* are based on *F*^2^, conventional *R*-factors *R* are based on *F*, with *F* set to zero for negative *F*^2^. The threshold expression of *F*^2^ > σ(*F*^2^) is used only for calculating *R*-factors(gt) *etc*. and is not relevant to the choice of reflections for refinement. *R*-factors based on *F*^2^ are statistically about twice as large as those based on *F*, and *R*-factors based on ALL data will be even larger.

### Fractional atomic coordinates and isotropic or equivalent isotropic displacement parameters (Å^2^)

**Table:**

	*x*	*y*	*z*	*U*_iso_*/*U*_eq_	
F1	0.28820 (7)	0.39132 (16)	0.58085 (8)	0.0963 (5)	
F2	0.66890 (12)	0.94956 (19)	0.80642 (12)	0.1445 (8)	
O1	0.57361 (8)	0.00028 (13)	0.57519 (7)	0.0679 (4)	
O2	0.66652 (8)	0.05261 (14)	0.50837 (8)	0.0695 (4)	
N1	0.72288 (11)	0.3120 (2)	0.51875 (9)	0.0692 (5)	
H1B	0.7509 (15)	0.380 (3)	0.5083 (10)	0.076 (7)*	
H1A	0.7197 (12)	0.239 (2)	0.4942 (11)	0.066 (6)*	
C1	0.49783 (15)	−0.1988 (2)	0.57203 (16)	0.0931 (8)	
H1C	0.4854	−0.2832	0.5484	0.140*	
H1D	0.4545	−0.1375	0.5734	0.140*	
H1E	0.5124	−0.2235	0.6154	0.140*	
C2	0.56045 (13)	−0.12602 (19)	0.53997 (12)	0.0746 (6)	
H2A	0.6054	−0.1850	0.5401	0.089*	
H2B	0.5473	−0.1043	0.4954	0.089*	
C3	0.62737 (10)	0.08577 (18)	0.55369 (10)	0.0578 (5)	
C4	0.63143 (11)	0.21328 (19)	0.58891 (10)	0.0572 (5)	
C5	0.67799 (11)	0.31852 (19)	0.56986 (10)	0.0572 (5)	
C6	0.68054 (11)	0.44920 (19)	0.60540 (10)	0.0609 (5)	
H6A	0.7040	0.5274	0.5867	0.073*	
C7	0.65069 (11)	0.4618 (2)	0.66361 (10)	0.0600 (5)	
C8	0.61518 (13)	0.3357 (2)	0.69326 (10)	0.0667 (5)	
H8A	0.5763	0.3668	0.7232	0.080*	
H8B	0.6532	0.2852	0.7181	0.080*	
C9	0.58026 (11)	0.23520 (19)	0.64523 (10)	0.0592 (5)	
H9A	0.5759	0.1435	0.6671	0.071*	
C10	0.50212 (11)	0.27746 (17)	0.62632 (9)	0.0559 (5)	
C11	0.44170 (12)	0.2002 (2)	0.64594 (11)	0.0684 (6)	
H11A	0.4499	0.1195	0.6709	0.082*	
C12	0.37006 (13)	0.2363 (2)	0.63060 (12)	0.0770 (6)	
H12A	0.3293	0.1813	0.6443	0.092*	
C13	0.35890 (12)	0.3530 (2)	0.59515 (11)	0.0690 (6)	
C14	0.41611 (13)	0.4322 (2)	0.57387 (12)	0.0748 (6)	
H14A	0.4070	0.5126	0.5490	0.090*	
C15	0.48779 (12)	0.3937 (2)	0.58902 (11)	0.0715 (6)	
H15A	0.5281	0.4476	0.5737	0.086*	
C16	0.65610 (11)	0.5913 (2)	0.70079 (11)	0.0648 (5)	
C17	0.66870 (14)	0.7180 (2)	0.67350 (13)	0.0781 (6)	
H17A	0.6753	0.7226	0.6288	0.094*	
C18	0.67220 (15)	0.8387 (3)	0.70790 (17)	0.0898 (8)	
H18A	0.6790	0.9256	0.6874	0.108*	
C19	0.66577 (16)	0.8309 (3)	0.77206 (18)	0.0979 (9)	
C20	0.6589 (2)	0.7078 (3)	0.80307 (16)	0.1065 (10)	
H20A	0.6580	0.7039	0.8481	0.128*	
C21	0.65327 (18)	0.5888 (3)	0.76701 (13)	0.0934 (8)	
H21A	0.6473	0.5023	0.7879	0.112*	

### Atomic displacement parameters (Å^2^)

**Table:**

	*U*^11^	*U*^22^	*U*^33^	*U*^12^	*U*^13^	*U*^23^
F1	0.0684 (8)	0.0980 (10)	0.1224 (13)	0.0017 (7)	−0.0188 (8)	−0.0061 (8)
F2	0.1599 (17)	0.0981 (12)	0.175 (2)	−0.0024 (11)	0.0216 (15)	−0.0716 (13)
O1	0.0700 (8)	0.0498 (7)	0.0838 (10)	−0.0096 (6)	0.0083 (7)	−0.0045 (6)
O2	0.0641 (8)	0.0553 (7)	0.0891 (11)	0.0019 (6)	0.0118 (8)	−0.0099 (7)
N1	0.0711 (11)	0.0588 (10)	0.0776 (13)	−0.0125 (8)	0.0186 (10)	−0.0101 (9)
C1	0.0954 (18)	0.0609 (12)	0.123 (2)	−0.0220 (12)	0.0001 (16)	0.0023 (13)
C2	0.0793 (14)	0.0475 (10)	0.0970 (17)	−0.0052 (9)	−0.0028 (12)	−0.0052 (10)
C3	0.0523 (9)	0.0474 (9)	0.0737 (13)	0.0016 (7)	−0.0012 (10)	0.0031 (8)
C4	0.0568 (10)	0.0500 (9)	0.0646 (12)	−0.0010 (8)	0.0013 (9)	0.0002 (8)
C5	0.0565 (10)	0.0524 (9)	0.0627 (12)	−0.0008 (8)	0.0039 (9)	−0.0009 (8)
C6	0.0622 (11)	0.0510 (9)	0.0696 (13)	−0.0085 (8)	0.0040 (10)	−0.0006 (8)
C7	0.0620 (11)	0.0568 (10)	0.0613 (12)	−0.0023 (8)	−0.0005 (9)	−0.0001 (8)
C8	0.0737 (13)	0.0658 (11)	0.0607 (12)	−0.0078 (9)	0.0050 (10)	0.0020 (9)
C9	0.0644 (11)	0.0490 (9)	0.0642 (12)	−0.0044 (8)	0.0068 (9)	0.0062 (8)
C10	0.0627 (11)	0.0452 (8)	0.0598 (11)	−0.0051 (8)	0.0086 (9)	−0.0033 (7)
C11	0.0690 (12)	0.0577 (11)	0.0784 (14)	−0.0072 (9)	0.0090 (11)	0.0090 (9)
C12	0.0653 (12)	0.0725 (13)	0.0932 (17)	−0.0160 (10)	0.0096 (12)	0.0049 (12)
C13	0.0622 (12)	0.0663 (12)	0.0786 (14)	0.0002 (9)	−0.0066 (11)	−0.0128 (10)
C14	0.0780 (14)	0.0578 (11)	0.0886 (16)	−0.0029 (10)	−0.0080 (12)	0.0086 (10)
C15	0.0680 (13)	0.0568 (11)	0.0897 (16)	−0.0090 (9)	0.0015 (11)	0.0141 (10)
C16	0.0651 (11)	0.0623 (11)	0.0671 (13)	0.0023 (9)	0.0036 (10)	−0.0047 (9)
C17	0.0852 (15)	0.0661 (13)	0.0831 (16)	−0.0069 (10)	0.0037 (12)	−0.0076 (11)
C18	0.0873 (17)	0.0642 (13)	0.118 (2)	−0.0003 (11)	0.0098 (16)	−0.0136 (14)
C19	0.0881 (18)	0.0780 (16)	0.128 (3)	0.0035 (13)	0.0108 (16)	−0.0422 (16)
C20	0.129 (3)	0.098 (2)	0.092 (2)	0.0000 (17)	0.0041 (18)	−0.0281 (16)
C21	0.119 (2)	0.0792 (15)	0.0816 (18)	−0.0014 (14)	0.0062 (15)	−0.0085 (13)

### Geometric parameters (Å, º)

**Table:**

F1—C13	1.360 (2)	C8—H8B	0.9900
F2—C19	1.355 (3)	C9—C10	1.519 (3)
O1—C3	1.350 (2)	C9—H9A	1.0000
O1—C2	1.445 (2)	C10—C11	1.382 (3)
O2—C3	1.230 (2)	C10—C15	1.393 (3)
N1—C5	1.348 (3)	C11—C12	1.375 (3)
N1—H1B	0.86 (3)	C11—H11A	0.9500
N1—H1A	0.88 (2)	C12—C13	1.366 (3)
C1—C2	1.491 (3)	C12—H12A	0.9500
C1—H1C	0.9800	C13—C14	1.359 (3)
C1—H1D	0.9800	C14—C15	1.381 (3)
C1—H1E	0.9800	C14—H14A	0.9500
C2—H2A	0.9900	C15—H15A	0.9500
C2—H2B	0.9900	C16—C17	1.369 (3)
C3—C4	1.438 (3)	C16—C21	1.397 (4)
C4—C5	1.376 (3)	C17—C18	1.372 (3)
C4—C9	1.518 (3)	C17—H17A	0.9500
C5—C6	1.466 (3)	C18—C19	1.359 (5)
C6—C7	1.345 (3)	C18—H18A	0.9500
C6—H6A	0.9500	C19—C20	1.360 (4)
C7—C16	1.478 (3)	C20—C21	1.379 (4)
C7—C8	1.508 (3)	C20—H20A	0.9500
C8—C9	1.536 (3)	C21—H21A	0.9500
C8—H8A	0.9900		
			
C3—O1—C2	117.35 (16)	C4—C9—H9A	106.6
C5—N1—H1B	121.5 (15)	C10—C9—H9A	106.6
C5—N1—H1A	118.0 (15)	C8—C9—H9A	106.6
H1B—N1—H1A	120 (2)	C11—C10—C15	117.13 (19)
C2—C1—H1C	109.5	C11—C10—C9	120.48 (17)
C2—C1—H1D	109.5	C15—C10—C9	122.39 (17)
H1C—C1—H1D	109.5	C12—C11—C10	122.20 (19)
C2—C1—H1E	109.5	C12—C11—H11A	118.9
H1C—C1—H1E	109.5	C10—C11—H11A	118.9
H1D—C1—H1E	109.5	C13—C12—C11	118.4 (2)
O1—C2—C1	106.7 (2)	C13—C12—H12A	120.8
O1—C2—H2A	110.4	C11—C12—H12A	120.8
C1—C2—H2A	110.4	C14—C13—F1	119.0 (2)
O1—C2—H2B	110.4	C14—C13—C12	122.1 (2)
C1—C2—H2B	110.4	F1—C13—C12	118.9 (2)
H2A—C2—H2B	108.6	C13—C14—C15	118.8 (2)
O2—C3—O1	120.91 (17)	C13—C14—H14A	120.6
O2—C3—C4	126.45 (17)	C15—C14—H14A	120.6
O1—C3—C4	112.64 (17)	C14—C15—C10	121.3 (2)
C5—C4—C3	120.69 (18)	C14—C15—H15A	119.3
C5—C4—C9	119.73 (17)	C10—C15—H15A	119.3
C3—C4—C9	119.46 (16)	C17—C16—C21	116.2 (2)
N1—C5—C4	124.37 (18)	C17—C16—C7	122.8 (2)
N1—C5—C6	115.42 (17)	C21—C16—C7	120.8 (2)
C4—C5—C6	120.21 (18)	C16—C17—C18	122.8 (3)
C7—C6—C5	122.06 (17)	C16—C17—H17A	118.6
C7—C6—H6A	119.0	C18—C17—H17A	118.6
C5—C6—H6A	119.0	C19—C18—C17	118.3 (3)
C6—C7—C16	122.23 (18)	C19—C18—H18A	120.8
C6—C7—C8	118.36 (18)	C17—C18—H18A	120.8
C16—C7—C8	119.27 (18)	F2—C19—C18	118.8 (3)
C7—C8—C9	114.13 (17)	F2—C19—C20	118.9 (3)
C7—C8—H8A	108.7	C18—C19—C20	122.3 (2)
C9—C8—H8A	108.7	C19—C20—C21	117.9 (3)
C7—C8—H8B	108.7	C19—C20—H20A	121.1
C9—C8—H8B	108.7	C21—C20—H20A	121.1
H8A—C8—H8B	107.6	C20—C21—C16	122.2 (3)
C4—C9—C10	113.29 (17)	C20—C21—H21A	118.9
C4—C9—C8	110.75 (16)	C16—C21—H21A	118.9
C10—C9—C8	112.54 (17)		
			
C3—O1—C2—C1	−178.43 (19)	C8—C9—C10—C15	70.1 (2)
C2—O1—C3—O2	−4.5 (3)	C15—C10—C11—C12	−1.0 (3)
C2—O1—C3—C4	175.00 (17)	C9—C10—C11—C12	179.0 (2)
O2—C3—C4—C5	5.9 (3)	C10—C11—C12—C13	−0.5 (4)
O1—C3—C4—C5	−173.59 (18)	C11—C12—C13—C14	1.2 (4)
O2—C3—C4—C9	−177.93 (19)	C11—C12—C13—F1	−178.6 (2)
O1—C3—C4—C9	2.6 (3)	F1—C13—C14—C15	179.4 (2)
C3—C4—C5—N1	−0.6 (3)	C12—C13—C14—C15	−0.5 (4)
C9—C4—C5—N1	−176.69 (19)	C13—C14—C15—C10	−1.1 (4)
C3—C4—C5—C6	178.74 (18)	C11—C10—C15—C14	1.8 (3)
C9—C4—C5—C6	2.6 (3)	C9—C10—C15—C14	−178.2 (2)
N1—C5—C6—C7	−166.7 (2)	C6—C7—C16—C17	22.1 (3)
C4—C5—C6—C7	14.0 (3)	C8—C7—C16—C17	−162.2 (2)
C5—C6—C7—C16	177.17 (19)	C6—C7—C16—C21	−153.7 (2)
C5—C6—C7—C8	1.4 (3)	C8—C7—C16—C21	22.1 (3)
C6—C7—C8—C9	−31.0 (3)	C21—C16—C17—C18	−5.4 (4)
C16—C7—C8—C9	153.08 (18)	C7—C16—C17—C18	178.6 (2)
C5—C4—C9—C10	96.8 (2)	C16—C17—C18—C19	2.7 (4)
C3—C4—C9—C10	−79.4 (2)	C17—C18—C19—F2	−179.8 (2)
C5—C4—C9—C8	−30.7 (3)	C17—C18—C19—C20	2.6 (5)
C3—C4—C9—C8	153.10 (17)	F2—C19—C20—C21	177.8 (3)
C7—C8—C9—C4	43.9 (2)	C18—C19—C20—C21	−4.5 (5)
C7—C8—C9—C10	−84.0 (2)	C19—C20—C21—C16	1.5 (5)
C4—C9—C10—C11	123.5 (2)	C17—C16—C21—C20	3.3 (4)
C8—C9—C10—C11	−109.9 (2)	C7—C16—C21—C20	179.4 (3)
C4—C9—C10—C15	−56.5 (2)		

### Hydrogen-bond geometry (Å, º)

**Table:**

*D*—H···*A*	*D*—H	H···*A*	*D*···*A*	*D*—H···*A*
N1—H1*B*···O2^i^	0.86 (3)	2.23 (3)	3.066 (2)	165 (2)
N1—H1*A*···O2	0.88 (2)	2.06 (2)	2.708 (2)	130.3 (19)
N1—H1*A*···F1^ii^	0.88 (2)	2.37 (2)	3.104 (2)	141.9 (19)

Symmetry codes: (i) −*x*+3/2, *y*+1/2, *z*; (ii) *x*+1/2, −*y*+1/2, −*z*+1.
